# The complete mitochondrial genome of *Osphronemus goramy* (Perciformes: Osphronemidae)

**DOI:** 10.1080/23802359.2016.1176885

**Published:** 2016-06-20

**Authors:** Pengfei Yan, Jing Fan

**Affiliations:** a College of Life Sciences, Leshan Normal University, Leshan, People’s Republic of China;; b The Ninth Middle School of Fengcheng, Fengcheng, People’s Republic of China

**Keywords:** Mitochondrial genome, Osphronemidae, *Osphronemus goramy*

## Abstract

The complete mitochondrial genome of *Osphronemus goramy* was sequenced and characterized in this study. It is 16,526 bp in length and contains 13 protein-coding genes, 22 transfer RNA genes, 2 ribosomal RNA genes and 1 non-coding control region (D-loop). The overall base composition of the heavy strand of *O. goramy* mtDNA is 29.64% A, 25.74% T, 30.11% C and 14.52% G. The phylogenetic result showed that there exists a close relationship among those genus within Anabantoidei. All results in this study provided an important data set for phylogenetic and taxonomic analyses of Osphronemus species.

The *Osphronemus goramy* (giant gourami) is a commercially important freshwater fish belonging to the family Osphronemidae, which was originated from Indonesian and has been introduced into other Asian countries (Pengseng & Boyd 2011; Azrita 2015). On account of its large size, beautiful appearance, tasty meat and easiness to conduct breeding operation, it is now considered to be an important species with high economic value (Bhimachar et al. 1944). The genus *Osphronemus* in the family Osphronemidae has five species (Froese & Pauly 2016), however, no complete mitogenome information of the genus is available in NCBI GenBank. We determined the mitochondrial genome (16,526 bp; GenBank accession no. KU984978) for this *Osphronemus* species in this study. The sequencing of complete mitochondrial genome of *O. goramy* would not only enrich mtDNA data for *Osphronemus*, but also useful for bringing out the fact of genetic divergence among the genus *Osphronemus*.

The samples of *O. goramy* were collected from an aquatic product market in Chengdu, Sichuan Province, China, and one individual was used to determine the mitochondrial genome and stored in Leshan Normal University (specimen number ZC160201). Standard phenol-chloroform method (Sambrook & Russell 2002) was used to extract genomic DNA from its tail fin clips. Ten primer sets were used to amplify overlapping segments. The PCR procedures followed from the study of Peng et al. (2007). PCR products were purified and then directly sequenced.

The overall base composition of the heavy strand of *O. goramy* mitochondrial genome is 29.64% A, 25.74% T, 30.11% C and 14.52% G, it consists of 2 ribosomal RNA genes, 13 protein-coding genes, 22 transfer RNA (tRNA) genes and 1 non-coding control regions (D-loop). Most of the genes were located in the heavy strand except ND6 and eight tRNA. As reported in vertebrates (Peng et al. 2007; Yan et al. 2016), the overall characteristics, including its overall organization and the gene arrangement pattern, are identical to that of the typical vertebrate mitogenome. All the protein-coding genes initiated with the traditional ATG start codon except for cox1. The cox1 gene started with GTG codon and was 1551 bp in length. Total length of the 13 protein-coding genes was 11,445 bp, which corresponded to 69.25% of the whole mitochondrial genome. The 12 protein-coding genes (with the exception of ND6) of *O. goramy* and other 11 species (Figure 1) in Perciformes downloaded from GenBank were aligned and analyzed using MEGA 5.0 (Tamura et al. 2011) and PAUP 4.0 (Swofford 2002). The phylogenetic result showed that *O. goramy* formed the same clade with other genus in Anabantoidei, and this indicated that they have close relationship. This result was in good agreement with the traditional ichthyological systematics and gave strong support to the basal position of *O. goramy* in Perciformes. This study provided an important data set for phylogenetic and taxonomic analyses of genus *Osphronemus* species.

**Figure 1.**
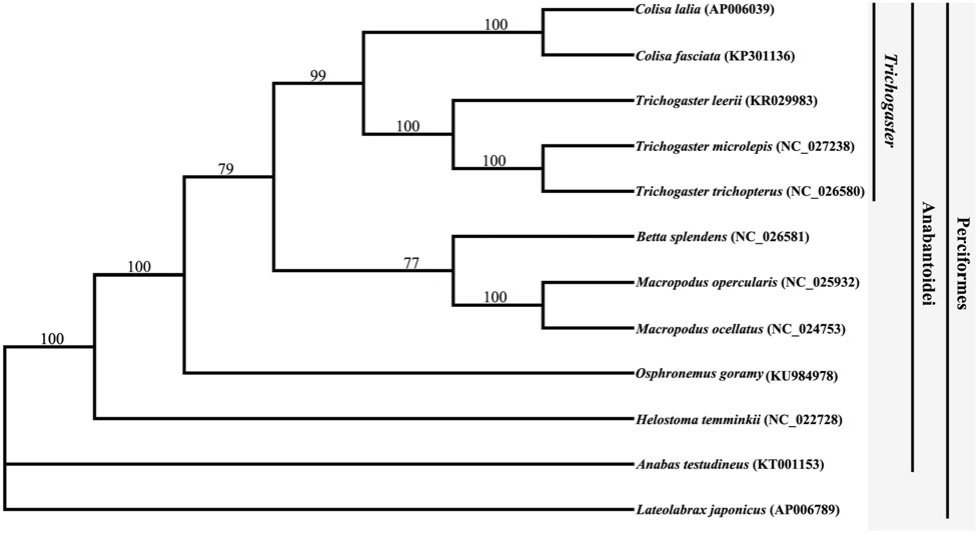
Bayesian tree reconstructed based on the sequences of 12 protein-coding genes (with the exception of ND6) from 12 species in Percoidea. The values beside the nodes are Bayesian posterior probabilities.
